# Immunogenicity and Safety of AS03‐Adjuvanted H7N9 Influenza Vaccine in Adults (18–64 and ≥65 Years): A Phase 1/2, Randomized, Placebo‐Controlled Trial

**DOI:** 10.1111/irv.70020

**Published:** 2024-12-19

**Authors:** Andrew Hastie, Tanya Clarke, Sophie Germain, Thierry Ollinger, Patricia Lese, Vinay Gupta

**Affiliations:** ^1^ GSK Rockville Maryland USA; ^2^ GSK Wavre Belgium; ^3^ GSK Bangalore India

**Keywords:** AS03, H7N9, influenza, vaccine

## Abstract

**Background:**

Influenza A/Hong Kong/125/2017 (H7N9) virus poses a pandemic risk owing to its evolving nature. This study evaluated the immunogenicity and safety of an AS03‐adjuvanted H7N9 vaccine in adults (18–64 years [younger] and ≥65 years [older]).

**Methods:**

Participants (younger, *n* = 418; older, *n* = 420) were randomized to receive one of six adjuvanted vaccines (hemagglutinin [1.9 μg, 3.75 μg, and 7.5 μg] with AS03_A_ or AS03_B_) or placebo. The co‐primary objectives were to determine whether the adjuvanted vaccines elicit an immune response against the vaccine‐homologous virus 21 days after the second vaccine dose and to evaluate the safety of the vaccines.

**Results:**

H7N9 AS03‐adjuvanted vaccines at various doses showed a humoral immune response but failed to meet CBER immunogenicity criteria. However, a trend of increased immune responses was observed with the AS03_A_ adjuvant versus the AS03_B_ adjuvant, particularly in older adults. In both age groups, injection site pain and fatigue occurred more frequently with adjuvanted vaccines. No reported serious adverse events were vaccine‐related.

**Conclusions:**

This study did not achieve its primary objective at any dose level. The modest immune response to AS03‐adjuvanted vaccines, consistent with other studies using similar antigens, highlights the need for continued research for H7N9 pandemic preparedness.

**Trial Registration:** NCT04789577 [ClinicalTrials.gov]

## 1 Introduction

Influenza A and B viruses cause most acute respiratory illnesses, with influenza A being the most virulent and the main driver of large epidemics [1]. The ongoing emergence of novel influenza A viruses, including the subtypes H5N1, H3N2, H9N2, A(H1N1) pdm09, H5N8, and influenza A/Hong Kong/125/2017 (H7N9), emphasizes the urgent need for continuous preparedness against future influenza pandemics [1, 2]. In February 2013, the H7N9 virus first emerged in Chinese poultry markets [3]. This virus poses a substantial threat of human influenza pandemics owing to its strong affinity for human‐type receptors and easy transmission to humans [4, 5, 6], as evidenced by the over 1560 human infections across five waves from February 2013 to September 2017 with a mortality rate of ~40%. Notably, between October 1, 2016 and September 30, 2017, 766 cases were reported in humans, constituting 48.9% of the total cases [6, 7, 8], raising concerns about the potential for a higher number of infections in the next wave. The H7N9 virus can cause rapidly progressing pneumonia with associated hypercytokinemia [9, 10]. Genetic changes in the H7N9 virus indicate mammalian adaptation, increasing the risk of human‐to‐human transmission [10, 11]. H7N9 and H7N1 influenza viruses can transmit through air in mammalian hosts (ferrets) without losing their virulence [11, 12, 13], highlighting the potential for H7 pandemics in humans and emphasizing the need for vaccine development. Although several clinically developed H7 inactivated and live‐attenuated influenza vaccines have limited immunogenicity in humans [10, 14, 15, 16], adjuvanted vaccines have demonstrated enhanced immunogenicity [17, 18, 19, 20]. In a mix‐and‐match study, an AS03‐adjuvanted monovalent A/Shanghai/2/2013(H7N9) vaccine elicited a more robust immune response than nonadjuvanted or MF59‐adjuvanted formulations when administered to adults in a two‐dose schedule [20]. This strain differs antigenically and temporally from the one used in our study, originating from a previous wave of H7N9 viruses.

Despite advancements in antiviral drugs, vaccination remains pivotal in influenza control and is a key component of the World Health Organization's pandemic preparedness. Immunosenescence particularly affects the elderly in terms of influenza‐related illness and death, highlighting the need for improved vaccines that boost both cellular and humoral immunity in older populations [21, 22]. Additionally, H7 vaccines have exhibited suboptimal immunogenicity in younger adults, but an adjuvant can significantly boost their effectiveness [20, 22]. Adjuvant use also supports antigen sparing, a critical consideration in pandemic vaccines. The US National Pre‐Pandemic Influenza Vaccine Stockpile includes diverse influenza vaccines, including those targeting influenza A(H5) and A(H7) subtypes, prepared with adjuvants (e.g., AS03) [23]. These adjuvants enhance immunogenicity and facilitate antigen sparing.

Clinical evidence for AS03‐containing antigen‐sparing formulations has demonstrated satisfactory immunogenicity in adults against potential pandemic subtypes, including H5N1, H1N1, H7N1, H7N9, and H9N2. Similar success has been observed for H5N1 and H1N1 in pediatric populations [24, 25]. As part of the US Department of Health and Human Services' pandemic preparedness activities, this study assessed the safety, immunogenicity, and antigen‐sparing ability of full and half doses of AS03 adjuvants combined with the H7N9 virus vaccine.

## 2 Materials and Methods

### 2.1 Participants, Vaccines, and Study Design

This phase 1/2 observer‐blinded, randomized, placebo‐controlled, multicenter trial included healthy males and nonpregnant females aged ≥18 years in the United States (eligibility criteria are detailed in Supporting Information Text S1). Ethical approval from independent committees obtained and ensured adherence to the Declaration of Helsinki, International Conference on Harmonization Good Clinical Practice guidelines, and relevant national regulations. All participants provided written informed consent. The inactivated, split‐virion vaccine developed using reverse genetics‐derived reassortant seed virus from H7N9 by World Health Organization Collaborating Centres and Reference Laboratories was manufactured by GSK Vaccines (Quebec, Canada). This vaccine was supplemented with AS03 as an oil‐in‐water emulsion containing 5.93 mg (AS03_B_) or 11.86 mg (AS03_A_) of DL‐alpha‐tocopherol. Participants were randomized using a blocking scheme with an allocation ratio of 1:1:1:1:1:1:1 to one of seven groups receiving two doses (doses 1 and 2) of hemagglutinin (HA) antigen doses (mixed with adjuvant [AS03_A_ or AS03_B_] in groups 1–6) or placebo, administered intramuscularly 21 days apart: (1) 1.9 μg HA/AS03_B_, (2) 1.9 μg HA/AS03_A_, (3) 3.75 μg HA/AS03_B_, (4) 3.75 μg HA/AS03_A_, (5) 7.5 μg HA/AS03_B_, (6) 7.5 μg HA/AS03_A_, or (7) phosphate‐buffered saline (placebo). HA antigen doses (1.9, 3.75, and 7.5 μg) were chosen based on specifications of the Biomedical Advanced Research and Development Authority.

### 2.2 Study Objectives and Endpoints

The primary immunogenicity objective was to evaluate whether the AS03_A_ or AS03_B_ adjuvanted H7N9 vaccines elicited an HA inhibition (HAI) antibody response to the vaccine‐homologous virus that met or exceeded the US Food and Drug Administration's Center for Biologics Evaluation and Research (CBER) immunogenicity criteria at the Day 43 visit. The primary immunogenicity objective was considered met if the following CBER criteria were fulfilled for any adjuvanted vaccine formulation: (1) For participants aged 18–64 years, the CBER immunogenicity criterion for seroconversion rate (SCR) was met if the lower limit of the 99.17% CI for SCR was ≥40%, and the CBER immunogenicity criterion for seroprotection rate (SPR) was met if the lower limit of the 99.17% CI for the incidence rate of reciprocal titers ≥40 in the HAI assay exceeded 70%. (2) For participants aged ≥65 years, the CBER immunogenicity criterion for SCR was met if the lower limit of the 99.17% CI for SCR was ≥30%, and the CBER immunogenicity criterion for SPR was met if the lower limit of the 99.17% CI for the incidence rate of reciprocal titers ≥40 in the HAI assay was ≥60%. The safety objective was to assess the safety and reactogenicity of vaccines up to day 43. Secondary objectives were to describe the vaccine‐homologous H7N9 HAI antibody profile in all study groups and to describe the vaccine‐homologous (H7N9) microneutralization (MN) antibody profiles in a subset of participants. The following derived parameters related to the tested vaccine virus were estimated for HAI titers: (1) geometric mean titers (GMTs) on Days 1, 22, and 43; (2) seropositivity rates on Days 1, 22, and 43; (3) SCR on Day 22; (4) SPR on Days 1 and 22; and (5) mean geometric increase (MGI) on Days 22 and 43. The following derived parameters related to the tested vaccine virus were estimated for MN titers: (1) GMTs on Days 1, 22, and 43; (2) seropositivity rates on Days 1, 22, and 43; and (3) vaccine response rate (VRR) on Days 22 and 43. The study procedure, including the definition of endpoints assessed, is detailed in the Supporting Information Text S2. Participants recorded solicited and unsolicited events on diary cards after each vaccination. Safety assessment procedures are described in the Supporting Information Text S3.

### 2.3 Statistical Analyses

Immunogenicity analyses were conducted in the per‐protocol cohorts, comprising participants who had adhered to the protocol, had received both vaccine doses, and had assay results for antibodies against the vaccine‐homologous HA antigen on both Days 22 and 43. Safety analyses were descriptive and conducted in the exposed set (total vaccinated cohort), including participants who had received at least one dose of the study vaccine or placebo. Statistical analyses are described in the Supporting Information Text S4.

## 3 Results

### 3.1 Participant Disposition and Characteristics

**FIGURE 1:**
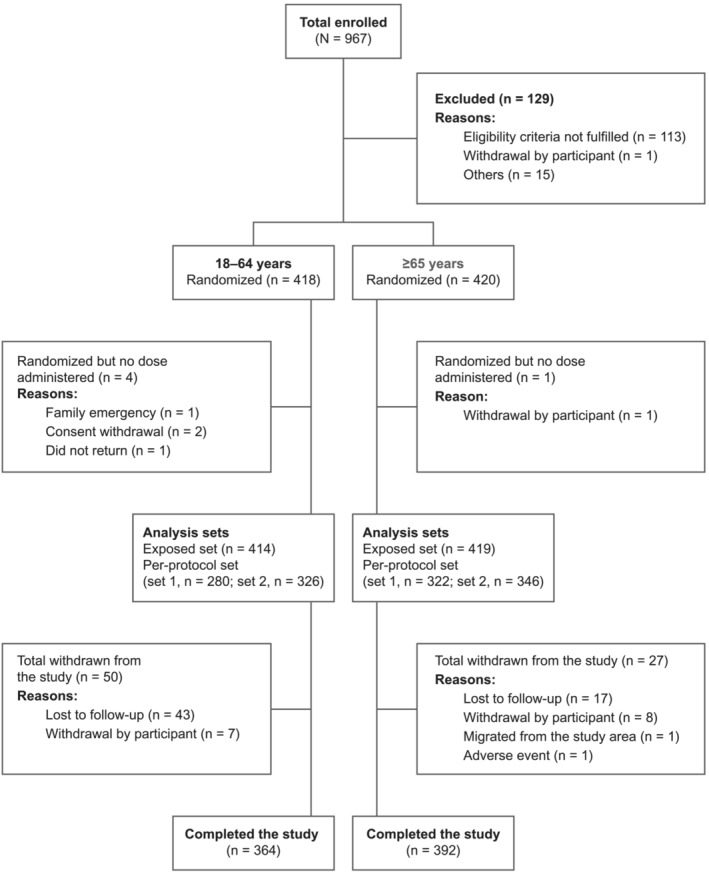
Study flowchart/CONSORT diagram.

Of the 967 participants screened, 838 were randomized to receive treatment, of whom 418 were aged 18–64 years (younger) and 420 were aged ≥65 years (older) (Figure 1). In the 18–64 years group, the mean age of participants was 44.8 years, 58.7% were women, and most (73.4%) were White. In the ≥65 years group, the mean age of participants was 72.1 years, 58.7% were women, and most (91.2%) were White. Demographics and baseline characteristics were generally similar across the study groups (Table 1).

**TABLE 1:** Participant demographics and baseline characteristics (exposed set).

	1.9 μg HA/AS03_B_	1.9 μg HA/AS03_A_	3.75 μg HA/AS03_B_	3.75 μg HA/AS03_A_	7.5 μg HA/AS03_B_	7.5 μg HA/AS03_A_	Placebo	Total
Age group: 18–64 years								
N	61	56	59	59	58	61	60	414
Age, years, mean (SD)	45.6 (11.46)	45.9 (12.32)	44.8 (12.23)	44.5 (13.05)	44.8 (13.05)	45.1 (12.03)	42.7 (13.53)	44.8 (12.49)
Sex, female, *n* (%)	30 (49.2)	32 (57.1)	37 (62.7)	35 (59.3)	42 (72.4)	32 (52.5)	35 (58.3)	243 (58.7)
BMI (kg/m^2^), mean (SD)	30.1 (7.72)	31.3 (8.13)	32.1 (7.30)	29.2 (6.52)	30.6 (8.30)	29.9 (6.46)	31.7 (8.81)	30.7 (7.65)
Race, *n* (%)								
Asian	1 (1.6)	1 (1.8)	2 (3.4)	2 (3.4)	1 (1.7)	4 (6.6)	0	11 (2.7)
Black or African American Native Hawaiian or Other	14 (23.0)	15 (26.8)	7 (11.9)	10 (16.9)	13 (22.4)	18 (29.5)	18 (30.0)	95 (22.9)
Pacific Islander	1 (1.6)	0	0	0	0	0	0	1 (0.2)
White	45 (73.8)	39 (69.6)	49 (83.1)	47 (79.7)	44 (75.9)	39 (63.9)	41 (68.3)	304 (73.4)
Other	0	1 (1.8)	1 (1.7)	0	0	0	1 (1.7)	3 (0.7)
Flu vaccination history in the past 3 years, yes, *n* (%)	41 (67.2)	32 (57.1)	39 (66.1)	42 (71.2)	43 (74.1)	35 (57.4)	33 (55.0)	265 (64.0)
Age group: ≥65 years								
N	60	60	60	60	59	60	60	419
Age, years, mean (SD)	72.6 (5.65)	71.2 (4.79)	72.2 (5.29)	72.4 (4.95)	72.3 (4.74)	71.7 (5.66)	72.2 (5.32)	72.1 (5.20)
Sex, female, *n* (%)	38 (63.3)	33 (55.0)	37 (61.7)	34 (56.7)	32 (54.2)	35 (58.3)	37 (61.7)	246 (58.7)
BMI (kg/m^2^), mean (SD)	29.0 (5.74)	29.6 (6.92)	30.4 (6.13)	28.7 (4.75)	29.3 (6.19)	30.0 (6.49)	30.7 (6.36)	29.7 (6.11)
Race, *n* (%)								
Asian	0	0	0	0	0	1 (1.7)	2 (3.3)	3 (0.7)
Black or African American	7 (11.7)	4 (6.7)	7 (11.7)	3 (5.0)	6 (10.2)	4 (6.7)	3 (5.0)	34 (8.1)
White	53 (88.3)	56 (93.3)	53 (88.3)	57 (95.0)	53 (89.8)	55 (91.7)	55 (91.7)	382 (91.2)
Flu vaccination history in the past 3 years, yes, *n* (%)	52 (86.7)	51 (85.0)	53 (88.3)	52 (86.7)	54 (91.5)	50 (83.3)	55 (91.7)	367 (87.6)

Abbreviations: BMI, body mass index; HA/AS03_A_, hemagglutinin adjuvanted with AS03_A_; HA/AS03_B_, hemagglutinin adjuvanted with AS03_B_; *N*, number of participants in each group; *n* (%): number (percentage) of individuals in each category.

### 3.2 Immunogenicity

#### 3.2.1 Seroprotection and SCRs Using Hemagglutinin Inhibition Assay

##### 3.2.1.1 Turkey Red Blood Cell (RBC) Hemagglutinin Inhibition Assay

At baseline (Day 1), SPR was 0 in both age groups, except with 1.9 μg HA/AS03_A_ in the younger age group (Table 2). After Dose 1 (Day 22), the turkey RBC HAI assay revealed a marginal increase in SPRs with higher doses (3.75 and 7.5 μg) in the 18–64 years group. In the ≥65 years group, SPR marginally increased with 1.9 μg HA/AS03_B_, 3.75 μg HA/AS03_A_, and both 7.5 μg HA/AS03_B_ and 7.5 μg HA/AS03_A_. In both age groups, SCR was the highest with 7.5 μg HA/AS03_A_.

**TABLE 2:** Summary of H7N9 HAI titer seropositivity rate, SPR, and SCR by age group and overall—Turkey RBC assay (per‐protocol set).

		1.9 μg HA/AS03_B_		1.9 μg HA/AS03_A_		3.75 μg HA/AS03_B_		3.75 μg HA/AS03_A_		7.5 μg HA/AS03_B_		7.5 μg HA/AS03_A_		Placebo	
		*N*	% (95% CI)	*N*	% (95% CI)	*N*	% (95% CI)	*N*	% (95% CI)	*N*	% (95% CI)	*N*	% (95% CI)	*N*	% (95% CI)
Age group: 18–64 years															
Seropositivity rate (%)	Day 1	47	2.1 (0.1, 11.3)	43	2.3 (0.1, 12.3)	48	0 (0.0, 7.4)	49	0 (0.0, 7.3)	47	0 (0.0, 7.5)	44	0 (0.0, 8.0)	48	0 (0.0, 7.4)
	Day 22	47	25.5 (13.9, 40.3)	43	30.2 (17.2, 46.1)	48	18.8 (8.9, 32.6)	49	32.7 (19.9, 47.5)	47	40.4 (26.4, 55.7)	44	52.3 (36.7, 67.5)	48	0 (0.0, 7.4)
	Day 43	46	80.4 (66.1, 90.6)	37	81.1 (64.8, 92.0)	40	80.0 (64.4, 90.9)	43	86.0 (72.1, 94.7)	38	78.9 (62.7, 90.4)	37	86.5 (71.2, 95.5)	39	0 (0.0, 9.0)
SPR (%)	Day 1	47	0 (0.0, 7.5)	43	2.3 (0.1, 12.3)	48	0 (0.0, 7.4)	49	0 (0.0, 7.3)	47	0 (0.0, 7.5)	44	0 (0.0, 8.0)	48	0 (0.0, 7.4)
	Day 22	47	0 (0.0, 7.5)	43	0 (0.0, 8.2)	48	10.4 (3.5, 22.7)	49	4.1 (0.5, 14.0)	47	10.6 (3.5, 23.1)	44	11.4 (3.8, 24.6)	48	0 (0.0, 7.4)
	Day 43	46	30.4 (14.4, 50.8)^a^	37	27.0 (10.6, 49.8)^a^	40	35.0 (16.8, 57.0)^a^	43	37.2 (19.1, 58.4)^a^	38	23.7 (8.6, 45.8)^a^	37	43.2 (22.6, 65.7)^a^	39	0 (0.0, 9.0)
SCR (%)	Day 22	47	0 (0.0, 7.5)	43	0 (0.0, 8.2)	48	10.4 (3.5, 22.7)	49	4.1 (0.5, 14.0)	47	10.6 (3.5, 23.1)	44	11.4 (3.8, 24.6)	48	0 (0.0, 7.4)
	Day 43	46	30.4 (14.4, 50.8)^a^	37	27.0 (10.6, 49.8)^a^	40	35.0 (16.8, 57.0)^a^	43	37.2 (19.1, 58.4)^a^	38	23.7 (8.6, 45.8)^a^	37	43.2 (22.6, 65.7)^a^	39	0 (0.0, 9.0)
Age group: ≥65 years															
Seropositivity rate (%)	Day 1	51	5.9 (1.2, 16.2)	50	6.0 (1.3, 16.5)	49	6.1 (1.3, 16.9)	45	6.7 (1.4, 18.3)	54	3.7 (0.5, 12.7)	50	8.0 (2.2, 19.2)	47	4.3 (0.5, 14.5)
	Day 22	51	11.8 (4.4, 23.9)	50	20.0 (10.0, 33.7)	49	28.6 (16.6, 43.3)	45	26.7 (14.6, 41.9)	54	25.9 (15.0, 39.7)	50	38.0 (24.7, 52.8)	47	6.4 (1.3, 17.5)
	Day 43	50	52.0 (37.4, 66.3)	46	73.9 (58.9, 85.7)	45	64.4 (48.8, 78.1)	40	72.5 (56.1, 85.4)	51	62.7 (48.1, 75.9)	47	80.9 (66.7, 90.9)	43	7.0 (1.5, 19.1)
SPR (%)	Day 1	51	0 (0.0, 7.0)	50	0 (0.0, 7.1)	49	0 (0.0, 7.3)	45	0 (0.0, 7.9)	54	0 (0.0, 6.6)	50	0 (0.0, 7.1)	47	0 (0.0, 7.5)
	Day 22	51	2.0 (0.0, 10.4)	50	0 (0.0, 7.1)	49	0 (0.0, 7.3)	45	4.4 (0.5, 15.1)	54	3.7 (0.5, 12.7)	50	8.0 (2.2, 19.2)	47	0 (0.0, 7.5)
	Day 43	50	24.0 (10.3, 43.0)^a^	46	28.3 (12.8, 48.5)^a^	45	13.3 (3.4, 31.7)^a^	40	15.0 (3.9, 35.1)^a^	51	15.7 (5.1, 33.2)^a^	47	38.3 (20.6, 58.5)^a^	43	0 (0.0, 8.2)
SCR (%)	Day 22	51	0 (0.0, 7.0)	50	0 (0.0, 7.1)	49	0 (0.0, 7.3)	45	4.4 (0.5, 15.1)	54	3.7 (0.5, 12.7)	50	8.0 (2.2, 19.2)	47	0 (0.0, 7.5)
	Day 43	50	24.0 (10.3, 43.0)^a^	46	28.3 (12.8, 48.5)^a^	45	13.3 (3.4, 31.7)^a^	40	15.0 (3.9, 35.1)^a^	51	15.7 (5.1, 33.2)^a^	47	38.3 (20.6, 58.5)^a^	43	0 (0.0, 8.2)

Abbreviations: %, percentage of responders; H7N9, influenza A/Hong Kong/125/2017 virus; HA/AS03_A_, hemagglutinin adjuvanted with AS03_A_; HA/AS03_B_, hemagglutinin adjuvanted with AS03_B_; HAI, hemagglutinin inhibition; *N*, number of participants in each group; RBC, red blood cell; SCR, seroconversion rate; SPR, seroprotection rate.

^a^ Reported as 99.17% CI.

Three weeks after dose 2 (day 43), the CBER criteria for SPR and SCR were not met across any of the dose groups and age groups (Table 2). In the 18–64 years group, SPR and SCR ranged from 23.7% to 43.2% with 7.5 μg HA/AS03_B_ and 7.5 μg HA/AS03_A_. In the ≥65 years group, SPR and SCR ranged from 13.3% to 38.3% with both 3.75 μg HA/AS03_B_ and 7.5 μg HA/AS03_A_.

##### 3.2.1.2 Horse RBC Hemagglutinin Inhibition Assay

At baseline (Day 1), SPR was 0% in both age groups, except with 1.9 μg HA/AS03_A_ in the older age group (Table 3). After dose 1 (Day 22), SPR increased marginally in both age groups. Both SPR and SCR were highest with 7.5 μg HA/AS03_B_ within the 18–64 years group and were relatively low across all dose groups within the ≥65 years group, with the highest (4.1%) recorded with 3.75 μg HA/AS03_B_.

**TABLE 3:** Participants with H7N9 HAI titer seropositivity rate, SPR, and SCR by age group and overall—Horse RBC HAI assay (per‐protocol set).

		1.9 μg HA/AS03_B_		1.9 μg HA/AS03_A_		3.75 μg HA/AS03_B_		3.75 μg HA/AS03_A_		7.5 μg HA/AS03_B_		7.5 μg HA/AS03_A_		Placebo	
		*N*	% (95% CI)	*N*	% (95% CI)	*N*	% (95% CI)	*N*	% (95% CI)	*N*	% (95% CI)	*N*	% (95% CI)	*N*	% (95% CI)
Age group: 18–64 years															
Seropositivity rate (%)	Day 1	47	0 (0.0, 7.5)	43	0 (0.0, 8.2)	48	0 (0.0, 7.4)	49	0 (0.0, 7.3)	47	0 (0.0, 7.5)	44	0 (0.0, 8.0)	48	0 (0.0, 7.4)
	Day 22	47	14.9 (6.2, 28.3)	43	9.3 (2.6, 22.1)	48	12.5 (4.7, 25.2)	49	16.3 (7.3, 29.7)	47	19.1 (9.1, 33.3)	44	15.9 (6.6, 30.1)	48	0 (0.0, 7.4)
	Day 43	46	71.7 (56.5, 84.0)	37	70.3 (53.0, 84.1)	40	65.0 (48.3, 79.4)	43	81.4 (66.6, 91.6)	38	60.5 (43.4, 76.0)	37	78.4 (61.8, 90.2)	39	0 (0.0, 9.0)
SPR (%)	Day 1	47	0 (0.0, 7.5)	43	0 (0.0, 8.2)	48	0 (0.0, 7.4)	49	0 (0.0, 7.3)	47	0 (0.0, 7.5)	44	0 (0.0, 8.0)	48	0 (0.0, 7.4)
	Day 22	47	0 (0.0, 7.5)	43	2.3 (0.1, 12.3)	48	10.4 (3.5, 22.7)	49	2.0 (0.1, 10.9)	47	10.6 (3.5, 23.1)	44	4.5 (0.6, 15.5)	48	0 (0.0, 7.4)
	Day 43	46	43.5 (24.7, 63.7)^a^	37	40.5 (20.4, 63.2)^a^	40	42.5 (22.7, 64.2)^a^	43	58.1 (37.2, 77.2)^a^	38	34.2 (15.8, 56.8)^a^	37	64.9 (42.0, 83.7)^a^	39	0 (0.0, 9.0)
SCR (%)	Day 22	47	0 (0.0, 7.5)	43	2.3 (0.1, 12.3)	48	10.4 (3.5, 22.7)	49	2.0 (0.1, 10.9)	47	10.6 (3.5, 23.1)	44	4.5 (0.6, 15.5)	48	0 (0.0, 7.4)
	Day 43	46	43.5 (24.7, 63.7)^a^	37	40.5 (20.4, 63.2)^a^	40	42.5 (22.7, 64.2)^a^	43	58.1 (37.2, 77.2)	38	34.2 (15.8, 56.8)^a^	37	64.9 (42.0, 83.7)^a^	39	0 (0.0, 9.0)
Age group: ≥65 years															
Seropositivity rate (%)	Day 1	51	0 (0.0, 7.0)	50	4.0 (0.5, 13.7)	49	0 (0.0, 7.3)	45	0 (0.0, 7.9)	54	0 (0.0, 6.6)	50	0 (0.0, 7.1)	47	2.1 (0.1, 11.3)
	Day 22	51	2.0 (0.0, 10.4)	50	8.0 (2.2, 19.2)	49	8.2 (2.3, 19.6)	45	8.9 (2.5, 21.2)	54	5.6 (1.2, 15.4)	50	16.0 (7.2, 29.1)	47	2.1 (0.1, 11.3)
	Day 43	50	40.0 (26.4, 54.8)	46	58.7 (43.2, 73.0)	45	44.4 (29.6, 60.0)	40	62.5 (45.8, 77.3)	51	51.0 (36.6, 65.2)	47	55.3 (40.1, 69.8)	43	2.3 (0.1, 12.3)
SPR (%)	Day 1	51	0 (0.0, 7.0)	50	4.0 (0.5, 13.7)	49	0 (0.0, 7.3)	45	0 (0.0, 7.9)	54	0 (0.0, 6.6)	50	0 (0.0, 7.1)	47	0 (0.0, 7.5)
	Day 22	51	0 (0.0, 7.0)	50	2.0 (0.1, 10.6)	49	4.1 (0.5, 14.0)	45	2.2 (0.1, 11.8)	54	1.9 (0.0, 9.9)	50	4.0 (0.5, 13.7)	47	2.1 (0.1, 11.3)
	Day 43	50	32.0 (16.1, 51.5)^a^	46	41.3 (22.9, 61.6)^a^	45	20.0 (7.2, 39.7)^a^	40	30.0 (13.1, 51.9)^a^	51	21.6 (8.8, 40.1)^a^	47	40.4 (22.4, 60.6)^a^	43	2.3 (0.1, 12.3)
SCR (%)	Day 22	51	0 (0.0, 7.0)	50	2.0 (0.1, 10.6)	49	4.1 (0.5, 14.0)	45	2.2 (0.1, 11.8)	54	1.9 (0.0, 9.9)	50	4.0 (0.5, 13.7)	47	0 (0.0, 7.5)
	Day 43	50	32.0 (16.1, 51.5)^a^	46	41.3 (22.9, 61.6)^a^	45	20.0 (7.2, 39.7)^a^	40	30.0 (13.1, 51.9)^a^	51	21.6 (8.8, 40.1)^a^	47	40.4 (22.4, 60.6)^a^	43	0 (0.0, 8.2)

Abbreviations: %, percentage of responders; H7N9, influenza A/Hong Kong/125/2017 virus; HA/AS03_A_, hemagglutinin adjuvanted with AS03_A_; HA/AS03_B_, hemagglutinin adjuvanted with AS03_B_; HAI, hemagglutinin inhibition; *N*, number of participants in each group; RBC, red blood cell; SCR, seroconversion rate; SPR, seroprotection rate.

^a^ Reported as 99.17% CI.

The CBER criteria for SPR and SCR were not met across any of the dose groups and age groups on day 43, except for SCR with 7.5 μg HA/AS03_A_ within the 18–64 years group. Notably, in the 18–64 years group that received 7.5 μg HA/AS03_A_, the lower limit of the 99.17% CI exceeded the CBER criteria for SCR, with an SCR [99.17% CI] of 64.9% [42.0, 83.7] on day 43. In the 18–64 years group, SPR and SCR ranged from 34.2% to 64.9% with both 7.5 μg HA/AS03_B_ and 7.5 μg HA/AS03_A_. In the ≥65 years group, SPR and SCR ranged from 20.0% to 41.3% with both 3.75 μg HA/AS03_B_ and 1.9 μg HA/AS03_A_.

#### 3.2.2 Geometric Mean Titers and MGI Using Hemagglutinin Inhibition and Microneutralization Assays

##### 3.2.2.1 Turkey RBC Hemagglutinin Inhibition Assay

At baseline (Day 1), GMTs were below the cutoff (<10) in both age groups (Table 4). After dose 1 (Day 22), GMTs marginally increased from baseline in both age groups, with the highest observed with 7.5 μg HA/AS03_A_. After Dose 2 (Day 43), GMTs ranged from 15.7 to 26.3 in the 18–64 years group and from 11.0 to 20.9 in the ≥65 years group. After dose 1 (Day 22), MGI ranged from 1.2 to 1.8 across all dose groups within the 18–64 years group and from 1.1 to 1.6 across all dose groups within the ≥65 years group.

**TABLE 4:** Summary of GMT and MGI for H7N9 HAI titer by age group and overall—Turkey RBC HAI assay (per‐protocol set).

		1.9 μg HA/AS03_B_		1.9 μg HA/AS03_A_		3.75 μg HA/AS03_B_		3.75 μg HA/AS03_A_		7.5 μg HA/AS03_B_		7.5 μg HA/AS03_A_		Placebo	
Age group: 18–64 years															
GMT		*N*	GMT (GSD) (95% CI)	*N*	GMT (GSD) (95% CI)	*N*	GMT (GSD) (95% CI)	*N*	GMT (GSD) (95% CI)	*N*	GMT (GSD) (95% CI)	*N*	GMT (GSD) (95% CI)	*N*	GMT (GSD) (95% CI)
	Day 1	47	5.1 (1.11) (4.9, 5.2)	43	5.2 (1.37) (4.8, 5.8)	48	5.0 (1.00) NC	49	5.0 (1.00) NC	47	5.0 (1.00) NC	44	5.0 (1.00) NC	48	5.0 (1.00) NC
	Day 22	47	6.2 (1.51) (5.5, 7.0)	43	6.3 (1.47) (5.6, 7.1)	48	7.3 (2.64) (5.5, 9.6)	49	7.2 (1.83) (6.1, 8.6)	47	9.0 (2.52) (6.9, 11.8)	44	9.2 (2.27) (7.2, 11.9)	48	5.0 (1.00) NC
	Day 43	46	16.7 (2.39) (12.9, 21.6)	37	15.7 (2.12) (12.2, 20.1)	40	18.6 (3.01) (13.0, 26.4)	43	20.7 (2.26) (16.1, 26.5)	38	16.2 (2.71) (11.7, 22.4)	37	26.3 (2.88) (18.5, 37.5)	39	5.0 (1.00) NC
MGI		N	MGI (GSD) (95% CI)	*N*	MGI (GSD) (95% CI)	*N*	MGI (GSD) (95% CI)	*N*	MGI (GSD) (95% CI)	*N*	MGI (GSD) (95% CI)	*N*	MGI (GSD) (95% CI)	*N*	MGI (GSD) (95% CI)
	Day 22	47	1.2 (1.54) (1.1, 1.4)	43	1.2 (1.48) (1.1, 1.4)	48	1.5 (2.64) (1.1, 1.9)	49	1.4 (1.83) (1.2, 1.7)	47	1.8 (2.52) (1.4, 2.4)	44	1.8 (2.27) (1.4, 2.4)	48	1.0 (1.00) NC
	Day 43	46	3.3 (2.39) (2.6, 4.3)	37	3.0 (2.25) (2.3, 3.9)	40	3.7 (3.01) (2.6, 5.3)	43	4.1 (2.26) (3.2, 5.3)	38	3.2 (2.71) (2.3, 4.5)	37	5.3 (2.88) (3.7, 7.5)	39	1.0 (1.00) NC

Age group: ≥65 years															
GMT		*N*	GMT (GSD) (95% CI)	*N*	GMT (GSD) (95% CI)	*N*	GMT (GSD) (95% CI)	*N*	GMT (GSD) (95% CI)	*N*	GMT (GSD) (95% CI)	*N*	GMT (GSD) (95% CI)	*N*	GMT (GSD) (95% CI)
	Day 1	51	5.3 (1.26) (4.9, 5.6)	50	5.2 (1.18) (5.0, 5.5)	49	5.2 (1.18) (5.0, 5.5)	45	5.2 (1.19) (5.0, 5.5)	54	5.2 (1.23) (4.9, 5.5)	50	5.3 (1.21) (5.0, 5.6)	47	5.1 (1.15) (4.9, 5.4)
	Day 22	51	5.6 (1.42) (5.1, 6.2)	50	5.7 (1.32) (5.3, 6.2)	49	6.4 (1.55) (5.7, 7.3)	45	6.8 (1.95) (5.6, 8.3)	54	6.5 (1.70) (5.7, 7.6)	50	8.6 (2.60) (6.6, 11.3)	47	5.2 (1.19) (5.0, 5.5)
	Day 43	50	11.6 (2.71) (8.7, 15.4)	46	15.6 (2.61) (11.8, 20.8)	45	11.0 (2.14) (8.7, 13.8)	40	12.7 (2.31) (9.7, 16.7)	51	11.5 (2.46) (8.9, 14.7)	47	20.9 (3.08) (15.0, 29.1)	43	5.2 (1.20) (5.0, 5.5)
MGI		*N*	MGI (GSD) (95% CI)	*N*	MGI (GSD) (95% CI)	*N*	MGI (GSD) (95% CI)	*N*	MGI (GSD) (95% CI)	*N*	MGI (GSD) (95% CI)	*N*	MGI (GSD) (95% CI)	*N*	MGI (GSD) (95% CI)
	Day 22	51	1.1 (1.26) (1.0, 1.1)	50	1.1 (1.28) (1.0, 1.2)	49	1.2 (1.50) (1.1, 1.4)	45	1.3 (1.88) (1.1, 1.6)	54	1.3 (1.62) (1.1, 1.4)	50	1.6 (2.57) (1.2, 2.1)	47	1.0 (1.11) (1.0, 1.0)
	Day 43	50	2.2 (2.68) (1.7, 2.9)	46	3.0 (2.65) (2.2, 4.0)	45	2.1 (2.08) (1.7, 2.6)	40	2.4 (2.29) (1.9, 3.2)	51	2.2 (2.40) (1.7, 2.8)	47	3.9 (3.05) (2.8, 5.5)	43	1.0 (1.11) (1.0, 1.0)

Abbreviations: GMT, geometric mean titer; GSD, geometric standard deviation; H7N9, influenza A/Hong Kong/125/2017 virus; HA/AS03_A_, hemagglutinin adjuvanted with AS03_A_; HA/AS03_B_, hemagglutinin adjuvanted with AS03_B_; HAI, hemagglutinin inhibition; MGI, mean geometric increase; *N*, number of participants in each group; NC, not calculated; RBC, red blood cell.

After dose 2 (day 43), MGI ranged from 3.0 to 5.3 in the 18–64 years group and from 2.1 to 3.9 in the ≥65 years group, with the highest observed with 7.5 μg HA/AS03_A_ in both age groups.

##### 3.2.2.2 Horse RBC Hemagglutinin Inhibition Assay

At baseline (day 1), GMTs were below the cutoff (<10) in both age groups (Table 5). After dose 1 (day 22), GMTs increased from baseline in both groups, with the highest observed with 7.5 μg HA/AS03_B_ in the 18–64 years group and with 7.5 μg HA/AS03_A_ in the ≥65 years group.

**TABLE 5:** Summary of GMT and MGI for H7N9 HAI titer by age group and overall—Horse RBC HAI assay (per‐protocol set).

		1.9 μg HA/AS03_B_		1.9 μg HA/AS03_A_		3.75 μg HA/AS03_B_		3.75 μg HA/AS03_A_		7.5 μg HA/AS03_B_		7.5 μg HA/AS03_A_		Placebo	
Age group: 18–64 years															
GMT		*N*	GMT (GSD) (95% CI)	*N*	GMT (GSD) (95% CI)	*N*	GMT (GSD) (95% CI)	*N*	GMT (GSD) (95% CI)	*N*	GMT (GSD) (95% CI)	*N*	GMT (GSD) (95% CI)	*N*	GMT (GSD) (95% CI)
	Day 1	47	5.0 (1.00) NC	43	5.0 (1.00) NC	48	5.0 (1.00) NC	49	5.0 (1.00) NC	47	5.0 (1.00) NC	44	5.0 (1.00) NC	48	5.0 (1.00) NC
	Day 22	47	5.8 (1.46) (5.2, 6.5)	43	5.6 (1.49) (5.0, 6.3)	48	7.0 (2.80) (5.2, 9.5)	49	6.3 (1.77) (5.3, 7.4)	47	7.3 (2.40) (5.7, 9.5)	44	6.6 (2.18) (5.2, 8.4)	48	5.0 (1.00) NC
	Day 43	46	19.7 (2.91) (14.3, 27.1)	37	19.9 (2.99) (13.8, 28.7)	40	18.5 (3.31) (12.7, 27.2)	43	28.4 (2.81) (20.6, 39.0)	38	16.8 (3.21) (11.4, 24.6)	37	31.7 (3.24) (21.5, 47.0)	39	5.0 (1.00) NC
MGI		*N*	MGI (GSD) (95% CI)	*N*	MGI (GSD) (95% CI)	*N*	MGI (GSD) (95% CI)	*N*	MGI (GSD) (95% CI)	*N*	MGI (GSD) (95% CI)	*N*	MGI (GSD) (95% CI)	*N*	MGI (GSD) (95% CI)
	Day 22	47	1.2 (1.46) (1.0, 1.3)	43	1.1 (1.49) (1.0, 1.3)	48	1.4 (2.80) (1.0, 1.9)	49	1.3 (1.77) (1.1, 1.5)	47	1.5 (2.40) (1.1, 1.9)	44	1.3 (2.18) (1.0, 1.7)	48	1.0 (1.00) NC
	Day 43	46	3.9 (2.91) (2.9, 5.4)	37	4.0 (2.99) (2.8, 5.7)	40	3.7 (3.31) (2.5, 5.4)	43	5.7 (2.81) (4.1, 7.8)	38	3.4 (3.21) (2.3, 4.9)	37	6.3 (3.24) (4.3, 9.4)	39	1.0 (1.00) NC

Age group: ≥65 years															
GMT		*N*	GMT (GSD) (95% CI)	*N*	GMT (GSD) (95% CI)	*N*	GMT (GSD) (95% CI)	*N*	GMT (GSD) (95% CI)	*N*	GMT (GSD) (95% CI)	*N*	GMT (GSD) (95% CI)	*N*	GMT (GSD) (95% CI)
	Day 1	51	5.0 (1.00) NC	50	5.5 (1.62) (4.8, 6.3)	49	5.0 (1.00) NC	45	5.0 (1.00) NC	54	5.0 (1.00) NC	50	5.0 (1.00) NC	47	5.1 (1.22) (4.9, 5.5)
	Day 22	51	5.1 (1.21) (4.9, 5.4)	50	5.7 (1.87) (4.8, 6.9)	49	5.6 (1.54) (4.9, 6.3)	45	5.8 (1.80) (4.9, 7.0)	54	5.4 (1.42) (4.9, 5.9)	50	6.5 (2.29) (5.1, 8.2)	47	5.2 (1.35) (4.8, 5.7)
	Day 43	50	12.1 (3.15) (8.7, 16.7)	46	18.0 (3.54) (12.4, 26.2)	45	10.9 (2.77) (8.0, 14.8)	40	15.6 (2.81) (11.2, 21.7)	51	11.6 (2.78) (8.7, 15.5)	47	17.9 (3.81) (12.1, 26.5)	43	5.2 (1.37) (4.8, 5.8)
MGI		*N*	MGI (GSD) (95% CI)	*N*	MGI (GSD) (95% CI)	*N*	MGI (GSD) (95% CI)	*N*	MGI (GSD) (95% CI)	*N*	MGI (GSD) (95% CI)	*N*	MGI (GSD) (95% CI)	*N*	MGI (GSD) (95% CI)
	Day 22	51	1.0 (1.21) (1.0, 1.1)	50	1.0 (1.73) (0.9, 1.2)	49	1.1 (1.54) (1.0, 1.3)	45	1.2 (1.80) (1.0, 1.4)	54	1.1 (1.42) (1.0, 1.2)	50	1.3 (2.29) (1.0, 1.6)	47	1.0 (1.11) (1.0, 1.0)
	Day 43	50	2.4 (3.15) (1.7, 3.3)	46	3.4 (3.39) (2.4, 4.9)	45	2.2 (2.77) (1.6, 3.0)	40	3.1 (2.81) (2.2, 4.3)	51	2.3 (2.78) (1.7, 3.1)	47	3.6 (3.81) (2.4, 5.3)	43	1.0 (1.11) (1.0, 1.0)

Abbreviations: GMT, geometric mean titer; GSD, geometric standard deviation; H7N9, influenza A/Hong Kong/125/2017 virus; HA/AS03_A_, hemagglutinin adjuvanted with AS03_A_; HA/AS03_B_, hemagglutinin adjuvanted with AS03_B_; HAI, hemagglutinin inhibition; MGI, mean geometric increase; *N*, number of participants in each group; NC, not calculated; RBC, red blood cell.

On Day 43, GMTs ranged from 16.8 to 31.7 in the 18–64 years group and from 10.9 to 18.0 in the ≥65 years group. On Day 22, MGI ranged from 1.1 to 1.5 across all dose groups within the 18–64 years group and from 1.0 to 1.3 across all dose groups within the ≥65 years group. On Day 43, MGI ranged from 3.4 to 6.3 in the 18–64 years group and from 2.2 to 3.6 in the ≥65 years group, with the highest observed with 7.5 μg HA/AS03_A_ in both age groups.

##### 3.2.2.3 Microneutralization Assay

On Day 43, GMTs increased from baseline across all age groups and dose levels, with the highest observed with 7.5 μg HA/AS03_A_ in the 18–64 years group (Table 6). Notably, GMTs were higher with doses containing AS03_A_ versus AS03_B_ at the 3.75 and 7.5 μg dose levels in both age groups. In the ≥65 years group, the highest GMT was recorded with 1.9 μg HA/AS03_A_.

**TABLE 6:** Summary of GMT for H7N9 MN titer by age group and overall (MN assay, per‐protocol set).

	1.9 μg HA/AS03_B_		1.9 μg HA/AS03_A_		3.75 μg HA/AS03_B_		3.75 μg HA/AS03_A_		7.5 μg HA/AS03_B_		7.5 μg HA/AS03_A_		Placebo	
Age group: 18–64 years	*N*	GMT (GSD)	*N*	GMT (GSD)	*N*	GMT (GSD)	*N*	GMT (GSD)	*N*	GMT (GSD)	*N*	GMT (GSD)	*N*	GMT (GSD)
		(95% CI)		(95% CI)		(95% CI)		(95% CI)		(95% CI)		(95% CI)		(95% CI)
Day 1	22	14.150 (1.0000) NC	22	14.150 (1.0000) NC	25	14.548 (1.1487) (13.7, 15.4)	26	14.150 (1.0000) NC	24	14.150 (1.0000) NC	22	14.150 (1.0000) NC	27	14.150 (1.0000) NC
Day 22	22	17.921 (1.6771) (14.2, 22.5)	22	16.564 (1.4423) (14.1, 19.5)	25	19.735 (3.1626) (12.3, 31.7)	26	18.472 (1.7455) (14.8, 23.1)	24	18.349 (1.8341) (14.2, 23.7)	22	19.087 (1.6190) (15.4, 23.6)	27	14.150 (1.0000) NC
Day 43	22	53.972 (2.9491) (33.4, 87.2)	19	45.462 (2.2111) (31.0, 66.6)	19	37.887 (2.9053) (22.7, 63.3)	22	53.967 (2.8710) (33.8, 86.1)	22	39.389 (2.5319) (26.1, 59.5)	18	84.768 (2.9191) (49.8, 144.4)	21	14.150 (1.0000) NC

Age group: ≥65 years	*N*	GMT (GSD) (95% CI)	*N*	GMT (GSD) (95% CI)	*N*	GMT (GSD) (95% CI)	*N*	GMT (GSD) (95% CI)	*N*	GMT (GSD) (95% CI)	*N*	GMT (GSD) (95% CI)	*N*	GMT (GSD) (95% CI)
Day 1	26	14.150 (1.0000) NC	24	14.150 (1.0000) NC	25	14.150 (1.0000) NC	25	14.150 (1.0000) NC	26	14.150 (1.0000) NC	26	14.532 (1.1456) (13.8, 15.4)	24	14.565 (1.1520) (13.7, 15.5)
Day 22	26	14.532 (1.1456) (13.8, 15.4)	24	14.991 (1.2162) (13.8, 16.3)	25	17.910 (1.5768) (14.8, 21.6)	25	16.030 (1.3472) (14.2, 18.1)	26	16.167 (1.5513) (13.5, 19.3)	26	17.514 (1.6014) (14.5, 21.2)	24	14.565 (1.1520) (13.7, 15.5)
Day 43	26	33.647 (2.3397) (23.9, 47.4)	22	40.016 (2.8823) (25.0, 64.0)	23	36.557 (2.4596) (24.8, 53.9)	24	31.760 (2.0990) (23.2, 43.4)	25	27.908 (1.9192) (21.3, 36.5)	24	38.876 (2.7343) (25.4, 59.4)	23	15.029 (1.2210) (13.8, 16.4)

Abbreviations: GMT, geometric mean titer; GSD, geometric standard deviation; H7N9, influenza A/Hong Kong/125/2017 virus; HA/AS03_A_, hemagglutinin adjuvanted with AS03_A_; HA/AS03_B_, hemagglutinin adjuvanted with AS03_B_; MN, microneutralization; *N*, number of participants in each group; NC, not calculated.

#### 3.2.3 Seropositivity Rates Using Hemagglutinin Inhibition and Microneutralization Assay

##### 3.2.3.1 Turkey RBC Hemagglutinin Inhibition Assay

At baseline (Day 1), in the 18–64 years group, seropositivity rates were 2.1% and 2.3% with 1.9 μg HA/AS03_B_ and 1.9 μg HA/AS03_A_, respectively, and 0% in the other dose groups. In the ≥65 years group, seropositivity rates ranged from 3.7% to 8.0% (Table 2). After dose 1 (Day 22), in both age groups, seropositivity rates increased from baseline across all dose groups, with the highest rate observed with 7.5 μg HA/AS03_B_ within both age groups.

Three weeks after dose 2 (day 43), seropositivity rates ranged from 78.9% to 86.5% in the 18–64 years group and from 52.0% to 80.9% in the ≥65 years group across all dose groups. Seropositivity rates were consistently higher with the AS03_A_‐adjuvanted vaccine versus the AS03_B_‐adjuvanted vaccine.

##### 3.2.3.2 Horse RBC Hemagglutinin Inhibition Assay

At baseline (day 1), the 18–64 years group had a 0% seropositivity rate across all dose groups, while the ≥65 years group showed seropositivity rates of 4.0% (1.9 μg HA/AS03_A_) and 2.1% (placebo group), with other dose groups at 0% (Table 3). On Day 22, in both age groups, seropositivity rates increased across all dose groups, with the highest observed with 7.5 μg HA/AS03_B_ within the 18–64 years group and with 7.5 μg HA/AS03_A_ within the ≥65 years group (Table 3).

On Day 43, seropositivity rates ranged from 60.5% to 81.4% in the 18–64 years group and from 40.0% to 62.5% in the ≥65 years group across all dose groups. In the 18–64 years group, seropositivity rates were higher with 3.75 μg HA/AS03_A_ and 7.5 μg HA/AS03_A_ compared with same doses of the AS03_B_ adjuvant. In the ≥65 years group, seropositivity rates were higher with AS03_A_ than with AS03_B_.

##### 3.2.3.3 Microneutralization Assay

On Day 22, seropositivity rates were the highest with 7.5 μg HA/AS03_A_ within the 18–64 years group and with 3.75 μg HA/AS03_B_ within the ≥65 years group (Table 7).

**TABLE 7:** Participants with H7N9 MN titer seropositivity rate and vaccine response rate by age group and overall (MN assay, per‐protocol set).

		1.9 μg HA/AS03_B_		1.9 μg HA/AS03_A_		3.75 μg HA/AS03_B_		3.75 μg HA/AS03_A_		7.5 μg HA/AS03_B_		7.5 μg HA/AS03_A_		Placebo	
		*N*	% (95% CI)	*N*	% (95% CI)	*N*	% (95% CI)	*N*	% (95% CI)	*N*	% (95% CI)	*N*	% (95% CI)	*N*	% (95% CI)
Age group: 18–64 years															
Seropositivity rate (%)	Day 1	22	0 (0.0, 15.4)	22	0 (0.0, 15.4)	25	4.0 (0.1, 20.4)	26	0 (0.0, 13.2)	24	0 (0.0, 14.2)	22	0 (0.0, 15.4)	27	0 (0.0, 12.8)
	Day 22	22	22.7 (7.8, 45.4)	22	18.2 (5.2, 40.3)	25	8.0 (1.0, 26.0)	26	23.1 (9.0, 43.6)	24	20.8 (7.1, 42.2)	22	31.8 (13.9, 54.9)	27	0 (0.0, 12.8)
	Day 43	22	72.7 (49.8, 89.3)	19	78.9 (54.4, 93.9)	19	73.7 (48.8, 90.9)	22	72.7 (49.8, 89.3)	22	68.2 (45.1, 86.1)	18	88.9 (65.3, 98.6)	21	0 (0.0, 16.1)
Vaccine response rate	Day 22	22	4.5 (0.1, 22.8)	22	4.5 (0.1, 22.8)	25	8.0 (1.0, 26.0)	26	11.5 (2.4, 30.2)	24	8.3 (1.0, 27.0)	22	9.1 (1.1, 29.2)	27	0 (0.0, 12.8)
	Day 43	22	54.5 (32.2, 75.6)	19	57.9 (33.5, 79.7)	19	31.6 (12.6, 56.6)	22	59.1 (36.4, 79.3)	22	45.5 (24.4, 67.8)	18	77.8 (52.4, 93.6)	21	0 (0.0, 16.1)
Age group: ≥65 years															
Seropositivity rate (%)	Day 1	26	0 (0.0, 13.2)	24	0 (0.0, 14.2)	25	0 (0.0, 13.7)	25	0 (0.0, 13.7)	26	0 (0.0, 13.2)	26	3.8 (0.1, 19.6)	24	4.2 (0.1, 21.1)
	Day 22	26	3.8 (0.1, 19.6)	24	8.3 (1.0, 27.0)	25	24.0 (9.4, 45.1)	25	16.0 (4.5, 36.1)	26	11.5 (2.4, 30.2)	26	19.2 (6.6, 39.4)	24	4.2 (0.1, 21.1)
	Day 43	26	61.5 (40.6, 79.8)	22	68.2 (45.1, 86.1)	23	65.2 (42.7, 83.6)	24	62.5 (40.6, 81.2)	25	64.0 (42.5, 82.0)	24	66.7 (44.7, 84.4)	23	8.7 (1.1, 28.0)
Vaccine response rate (%)	Day 22	26	0 (0.0, 13.2)	24	0 (0.0, 14.2)	25	8.0 (1.0, 26.0)	25	0 (0.0, 13.7)	26	3.8 (0.1, 19.6)	26	7.7 (0.9, 25.1)	24	0 (0.0, 14.2)
	Day 43	26	38.5 (20.2, 59.4)	22	36.4 (17.2, 59.3)	23	43.5 (23.2, 65.5)	24	37.5 (18.8, 59.4)	25	24.0 (9.4, 45.1)	24	41.7 (22.1, 63.4)	23	0 (0.0, 14.8)

Abbreviations: %, percentage of responders; H7N9, influenza A/Hong Kong/125/2017 virus; HA/AS03_A_, hemagglutinin adjuvanted with AS03_A_; HA/AS03_B_, hemagglutinin adjuvanted with AS03_B_; MN, microneutralization; *N*, number of participants in each group.

On Day 43, seropositivity rates increased, with the highest rates recorded with 7.5 μg HA/AS03_A_ within the 18–64 years group and with 1.9 μg HA/AS03_A_ within the ≥65 years group.

#### 3.2.4 Vaccine Response Rate Using Microneutralization Assay

After Dose 1 (day 22), VRR ranged from 4.5% (1.9 μg HA/AS03_B_ and 1.9 μg HA/AS03_A_) to 11.5% (3.75 μg HA/AS03_A_) in the 18–64 years group. VRRs were slightly lower in the ≥65 years group, ranging from 0% (1.9 μg HA/AS03_B_, 1.9 μg HA/AS03_A_, and 3.75 μg HA/AS03_A_) to 8.0% (3.75 μg HA/AS03_B_; Table 7).

After Dose 2 (Day 43), in the 18–64 years group, VRR ranged from 31.6% to 77.8% with both 3.75 μg HA/AS03_B_ and 7.5 μg HA/AS03_A_. In the ≥65 years group, VRR ranged from 24.0% to 43.5% with both 7.5 μg HA/AS03_B_ and 3.75 μg HA/AS03_B_. After both doses, VRR in the MN assay was higher in both age groups for AS03_A_ than for AS03_B_.

### 3.3 Safety Analysis

A summary of adverse events (AEs) is presented in Table 8. No clinically significant differences were observed among the study groups. Overall, AS03_A_ showed a higher incidence of solicited administration site events than AS03_B_. Pain was the most common solicited administration site event, and fatigue was the predominant systemic AE in both age groups. The ≥65 years group generally reported fewer events. Common vaccine‐related unsolicited AEs included headache, injection site pain, fatigue, and diarrhea. Serious AEs (SAEs) occurred in 4.6% of the participants, with atrial fibrillation, pneumonia, transitional cell carcinoma, and femur fracture being the most frequently reported. No SAEs were vaccine‐related, and no clustering by dose level occurred. No potential immune‐mediated disorders were reported until Day 43. No deaths occurred during the study.

**TABLE 8:** Safety outcomes reported by age group (exposed set).

	1.9 μg HA/AS03_B_	1.9 μg HA/AS03_A_	3.75 μg HA/AS03_B_	3.75 μg HA/AS03_A_	7.5 μg HA/AS03_B_	7.5 μg HA/AS03_A_	Placebo	Total
Age group: 18–64 years								
*N* (after second vaccination)	54	48	55	51	50	52	55	365
Solicited AEs within 7 days postvaccination								
Injection site AEs (after second vaccination, any severity), *n* (%)								
Pain	31 (57.4)	32 (66.7)	33 (60.0)	40 (78.4)	24 (48.0)	33 (63.5)	4 (7.3)	197 (54.0)
Redness (>20 mm)	1 (1.9)	0	0	2 (3.9)	0	1 (1.9)	0	4 (1.1)
Swelling (>20 mm)	1 (1.9)	4 (8.3)	1 (1.8)	4 (7.8)	0	2 (3.8)	0	12 (3.3)
General AEs (after second vaccination, any severity), *n* (%)								
Fatigue	17 (31.5)	12 (25.0)	20 (36.4)	18 (35.3)	11 (22.0)	21 (40.4)	10 (18.2)	109 (29.9)
Headache	16 (29.6)	13 (27.1)	14 (25.5)	15 (29.4)	9 (18.0)	16 (30.8)	5 (9.1)	88 (24.1)
Muscle ache	8 (14.8)	7 (14.6)	11 (20.0)	11 (21.6)	4 (8.0)	10 (19.2)	1 (1.8)	52 (14.2)
Joint pain	4 (7.4)	8 (16.7)	8 (14.5)	8 (15.7)	6 (12.0)	9 (17.3)	5 (9.1)	48 (13.2)
Shivering (chills)	4 (7.4)	3 (6.3)	5 (9.1)	2 (3.9)	1 (2.0)	4 (7.7)	0	19 (5.2)
Sweating	4 (7.4)	4 (8.3)	4 (7.3)	3 (5.9)	4 (8.0)	4 (7.7)	3 (5.5)	26 (7.1)
Nausea	3 (5.6)	6 (12.5)	6 (10.9)	3 (5.9)	4 (8.0)	6 (11.5)	3 (5.5)	31 (8.5)
Vomiting	0	4 (8.3)	0	0	0	0	1 (1.8)	5 (1.4)
Diarrhea	2 (3.7)	5 (10.4)	4 (7.3)	4 (7.8)	4 (8.0)	2 (3.8)	3 (5.5)	24 (6.6)
Abdominal pain	2 (3.7)	1 (2.1)	6 (10.9)	2 (3.9)	1 (2.0)	3 (5.8)	2 (3.6)	17 (4.7)
Fever	0	2 (4.2)	0	1 (2.0)	0	1 (1.9)	0	4 (1.1)
Unsolicited AEs within 21 days postvaccination, *n* (%)								
In at least 1 participant	21 (34.4)	17 (30.4)	18 (30.5)	16 (27.1)	15 (25.9)	26 (42.6)	14 (23.3)	127 (30.7)
At least 1 related Unsolicited AEs	11 (18.0)	6 (10.7)	6 (10.2)	4 (6.8)	8 (13.8)	13 (21.3)	6 (10.0)	54 (13.0)
Medically attended AEs up to 21 days after each vaccination, *n* (%)								
In at least 1 participant	4 (6.6)	5 (8.9)	7 (11.9)	5 (8.5)	0	8 (13.1)	2 (3.3)	31 (7.5)
SAEs occurring in at least 1 participant, *n* (%)	1 (1.6)	1 (1.8)	5 (8.5)	0	0	2 (3.3)	2 (3.3)	11 (2.7)
Age group: ≥65 years								
N (Second vaccination)	58	59	57	57	56	53	56	396
Solicited AEs within 7 days postvaccination								
Injection site AEs (after second vaccination, any severity), *n* (%)								
Pain	22 (37.9)	30 (50.8)	25 (43.9)	26 (45.6)	18 (32.1)	23 (43.4)	9 (16.1)	153 (38.6)
Redness (>20 mm)	0	3 (5.1)	0	1 (1.8)	1 (1.8)	1 (1.9)	0	6 (1.5)
Swelling (>20 mm)	3 (5.2)	4 (6.8)	0	1 (1.8)	1 (1.8)	2 (3.8)	0	11 (2.8)
General AEs (after second vaccination, any severity), *n* (%)								
Fatigue	17 (29.3)	8 (13.6)	8 (14.0)	11 (19.3)	10 (17.9)	13 (24.5)	10 (17.9)	77 (19.4)
Headache	7 (12.1)	6 (10.2)	5 (8.8)	5 (8.8)	7 (12.5)	5 (9.4)	7 (12.5)	42 (10.6)
Muscle ache	6 (10.3)	7 (11.9)	7 (12.3)	4 (7.0)	5 (8.9)	6 (11.3)	1 (1.8)	36 (9.1)
Joint pain	4 (6.9)	2 (3.4)	7 (12.3)	4 (7.0)	4 (7.1)	5 (9.4)	1 (1.8)	27 (6.8)
Shivering (chills)	5 (8.6)	1 (1.7)	1 (1.8)	1 (1.8)	1 (1.8)	2 (3.8)	1 (1.8)	12 (3.0)
Sweating	2 (3.4)	0	3 (5.3)	4 (7.0)	2 (3.6)	4 (7.5)	2 (3.6)	17 (4.3)
Nausea	3 (5.2)	2 (3.4)	4 (7.0)	3 (5.3)	3 (5.4)	1 (1.9)	2 (3.6)	18 (4.5)
Vomiting	1 (1.7)	1 (1.7)	0	1 (1.8)	0	0	0	3 (0.8)
Diarrhea	3 (5.2)	0	5 (8.8)	3 (5.3)	2 (3.6)	2 (3.8)	4 (7.1)	19 (4.8)
Abdominal pain	3 (5.2)	0	4 (7.0)	3 (5.3)	4 (7.1)	2 (3.8)	3 (5.4)	19 (4.8)
Fever	1 (1.7)	0	0	1 (1.8)	0	0	0	2 (0.5)
Unsolicited AEs within 21 days postvaccination, *n* (%)								
In at least 1 participant	19 (31.7)	15 (25.0)	15 (25.0)	18 (30.0)	15 (25.4)	13 (21.7)	19 (31.7)	114 (27.2)
At least 1 related Unsolicited AEs	1 (1.7)	6 (10.0)	4 (6.7)	7 (11.7)	7 (11.9)	4 (6.7)	8 (13.3)	37 (8.8)
Medically attended AEs up to 21 days after each vaccination, *n* (%)								
In at least 1 participant	6 (10.0)	7 (11.7)	7 (11.7)	7 (11.7)	5 (8.5)	8 (13.3)	5 (8.3)	45 (10.7)
SAEs occurring in at least 1 participant, *n* (%)	2 (3.3)	2 (3.3)	5 (8.3)	4 (6.7)	6 (10.2)	3 (5.0)	5 (8.3)	27 (6.4)

Abbreviations: AE, adverse event; HA/AS03_A_, hemagglutinin adjuvanted with AS03_A_; HA/AS03_B_, hemagglutinin adjuvanted with AS03_B_; *N*, number of participants in each group; *n* (%): number (percentage) of individuals in each category; SAE, serious adverse event.

## 4 Discussion

The findings from this phase 1/2 randomized, placebo‐controlled trial indicate that none of the administered doses of the H7N9 AS03‐adjuvanted vaccine was able to meet CBER's immunogenicity criteria for influenza vaccine in both participants aged 18–64 years and those aged ≥65 years. Overall, the primary immunogenicity objective was not achieved, as none of the doses in the HAI assay (using turkey and horse RBCs) yielded an SPR or SCR that met the specified criteria outlined by the CBER, in either age group. Of note, in the horse RBC HAI assay, the 7.5 μg HA/AS03_A_ dose group within the 18–64 years group met the CBER criteria for SCR. Specifically, the lower limit of the 99.17% CI exceeded the 40% threshold on Day 43, with a value of 42.0%. Comparatively, SPR and SCR were lower in the ≥65 years group than in the 18–64 years group at each dose level. In the HAI assay, the increase in seropositivity rates was minimal following Dose 1 but notably greater after Dose 2, mainly due to the low number of participants with baseline seropositivity, necessitating two doses to trigger an immune response. In the turkey and horse RBC HAI assays, after wo doses, the AS03_A_‐adjuvanted vaccine exhibited higher seropositivity rates than the AS03_B_‐adjuvanted vaccine. Moreover, the immune responses were lower in all dose groups within the ≥65 years group than within the 18–64 years group. Evidence of an antigen dose response was inconsistent in either adjuvant group; however, the highest response was often observed with 7.5 μg HA/AS03_A_ within either age group. Our findings align with those of another randomized clinical trial that demonstrated suboptimal immunogenicity of the AS03‐adjuvanted 2017 inactivated influenza A/H7N9 vaccine (H7N9 IIV) after a single dose, with only a modest improvement after administration of two doses [26]. Additionally, our observations revealed a similar degree of modest immunogenicity to that observed for the 2017 H7N9 IIV, when used in conjunction with the AS03 adjuvant (NCT03312231), and substantially higher than unadjuvanted 2017 H7N9 IIV. The immunogenicity induced by AS03‐adjuvanted 2017 H7N9 IIV was lower than that induced by AS03‐adjuvanted 2013 H7N9 IIV at the same dosage [20]. This can be attributed to the presence of immune‐escape variants in poultry, allowing the virus to evade vaccine‐induced immunity, posing a significant challenge to the development of effective H7N9 influenza vaccines [27]. This is crucial because vaccination remains the primary method for controlling H7N9 pandemics. The modest response to the vaccine in this study strongly emphasizes the need for further research to enhance our preparedness for potential future H7N9 pandemics.

In this study, GMTs were typically lower in the older group than in the younger group in horse and turkey RBC HAI assays. Additionally, groups receiving AS03_A_ tended to demonstrate higher GMTs in both turkey and horse RBC HAI assays. Moreover, the MN assay revealed higher overall VRRs in groups receiving AS03_A_ versus AS03_B_. While there was some evidence of an antigen dose response in the AS03_A_ groups, this was not evident in the AS03_B_ groups. Furthermore, VRRs were slightly lower in the older group than in the younger group. The potency of the AS03 adjuvant (11.86 or 5.93 mg tocopherol in AS03_A_ or AS03_B_, respectively) appears to exert a more pronounced influence on immunogenicity than the antigen content. This observation aligns with findings from a study involving an H7N9 HA antigen produced by a different manufacturer mixed with GSK's AS03_A_ at the point of use [20] and a study of the AS03‐adjuvanted H5N1 pandemic vaccine [28]. Furthermore, our observation of lower immune responses in older versus younger age groups is consistent with findings of a previous study that indicated generally lower immune responses in older participants (aged 41–64 years) than in younger participants (aged 18–40 years) [19, 20, 29, 30].

Overall, the H7N9 AS03‐adjuvanted vaccine demonstrated favorable tolerability. Injection site pain was the most reported solicited symptom, consistent with results of a previous study involving adjuvanted influenza vaccines [10]. The tolerability profile was deemed acceptable, given that most participants returned for their second vaccine dose. Most AEs were of low severity and resolved spontaneously. None of the SAEs were attributed to vaccination. In this study, a trend indicating higher reactogenicity associated with AS03_A_‐adjuvanted vaccines compared with AS03_B_ formulations was observed. However, all formulations were well tolerated, and no safety concerns emerged during the study. The AS03_A_‐adjuvanted formulations elicited an enhanced immune response when compared with the AS03_B_‐adjuvanted formulations. Thus, the clinical risk–benefit ratio of using a high‐potency adjuvant appears acceptable. It is worth emphasizing that participants aged ≥65 years tended to experience fewer AEs than those aged 18–64 years.

Immunogenicity was evaluated based solely on HAI and MN assays. No additional assessments, such as evaluating immunity at the cellular level, were conducted, even though they could provide more insights. However, regulatory standards for accepting such assessments have not been set. No formal comparisons were conducted between the vaccine groups with adjuvants; instead, the analyses were presented descriptively. These aspects could be limitations of this study.

## 5 Conclusion

This study did not achieve its primary objectives at any dose level. However, a trend of increased immune responses with the AS03_A_ adjuvant compared with the AS03_B_ adjuvant was observed. The modest immune response observed in this study emphasizes the need for continued research to enhance preparedness for future H7N9 pandemics. Importantly, the absence of safety concerns provides confidence for future research.

## Author Contributions

**Andrew Hastie:** conceptualization, methodology, formal analysis, writing – review and editing, funding acquisition. **Tanya Clarke:** conceptualization, methodology, writing – review and editing, formal analysis. **Sophie Germain:** conceptualization, methodology, writing – review and editing, formal analysis. **Thierry Ollinger:** conceptualization, methodology, writing – review and editing, formal analysis. **Patricia Lese:** conceptualization, methodology, writing – review and editing, formal analysis. **Vinay Gupta:** conceptualization, methodology, formal analysis, writing – review and editing.

## Ethics Statement

Ethical approval from Institutional Review Boards (IRB) obtained and ensured adherence to the Declaration of Helsinki, International Conference on Harmonization Good Clinical Practice guidelines, and relevant national regulations.

## Consent

Written informed consent or assent was obtained from all individual participants included in this study or their parent(s), guardian(s), or legally acceptable representative(s) before participation in the study.

## Conflicts of Interest

A. H., T. C., S. G., T. O., P. L., and V. G. are employees of the GSK group of companies. A. H. and T. C possess stock options in GSK.

## Permission to Reproduce Material From Other Sources

Not applicable.

## Trademark Statement

AS03 is a trademark owned by or licensed to the GSK group of companies.

## Supporting information


**Data S1** Supporting Information.

## Data Availability

Anonymized individual participant data and study documents can be requested for further research from http://www.gsk‐studyregister.com/en/.
