# Supplementary material for: Immunogenicity and Safety of AS03‐Adjuvanted H7N9 Influenza Vaccine in Adults (18–64 and ≥65 Years): A Phase 1/2, Randomized, Placebo‐Controlled Trial
Source: Influenza Other Respir Viruses. 2024 Dec 19;18(12):e70020. doi: 10.1111/irv.70020 (PMC11658966; doi:10.1111/irv.70020)
Supplement: Supplementary file 1 — Data S1 Supporting Information. [file IRV-18-e70020-s001.docx]

**Supplementary Text**

**Supplementary Text 1**

**Inclusion Criteria**

1. Healthy individuals, as determined by medical history and prestudy clinical examination.
2. Male or female participants aged ≥18 years at the first vaccination.
3. Participants capable of and willing to comply with protocol requirements.
4. Written or witnessed/thumb-printed informed consent obtained before the start of any study-specific procedure.
5. Female participants of nonchildbearing potential or childbearing potential meeting the contraception criteria.

**Exclusion Criteria**

*Medical Conditions*

1. Current or history of autoimmune disorder(s).
2. History of hypersensitivity to vaccine components or latex.
3. Acute or chronic clinically significant uncontrolled pulmonary, cardiovascular, hepatic, or renal abnormalities.
4. Confirmed or suspected immunosuppressive or immunodeficiency condition.
5. Recurrent or uncontrolled neurological disorders, seizures, or a history of Guillain-Barré syndrome.
6. Diagnosis or history of narcolepsy, cancer within the past 3 years, HIV-positive status, or significant hematological abnormalities.
7. Bedridden participants or any clinical condition posing additional risk, per the investigator’s judgment.

*Prior/Concomitant Therapy*

1. Use of investigational or nonregistered products within 30 days before the first dose.
2. Administration of long-acting immune-modifying drugs, immunoglobulins, blood products, or plasma derivatives.
3. Chronic use of immunosuppressants exceeding 14 days or prednisone equivalent ≥20 mg/day.

*Prior/Concurrent Clinical Study Experience*

1. Participation in another study with exposure to investigational/noninvestigational products during the study period.

*Other Exclusion Criteria*

1. Pregnant/lactating females or those planning pregnancy/discontinuing contraception within 2 months postvaccination.
2. History of/current chronic alcohol/drug consumption or abuse.
3. Any condition posing additional risks, per the investigator’s judgment.
4. Study personnel, immediate dependents, or family or household members.

**Supplementary Text 2**

The immunogenicity measures (hemagglutination inhibition [HAI] and microneutralization [MN]) employed in this study were selected based on scientific consensus and are considered appropriate for describing the immune response against the influenza A/Hong Kong/125/2017 (H7N9) vaccine in this study. The primary endpoint analysis utilized data from the turkey red blood cell (RBC) HAI assay, while the horse RBC HAI assay data were used as supporting data or for secondary analysis. Blood samples for immunogenicity analyses were collected on days 1, 22, and 43. Immunogenicity was assessed by HAI and MN assays. HAI antibody titers were assessed using turkey RBCs and horse RBCs. To eliminate nonspecific inhibitors of hemagglutination in the HAI assay, serum samples were pretreated with receptor-destroying enzyme (RDE). For the horse RBC HAI assay, to eliminate nonspecific agglutinins to horse RBCs, serum samples were pre-incubated with horse RBCs in addition to the RDE pretreatment. Madin-Darby canine kidney cells and enzyme-linked immunosorbent assay–based detection was used to assess MN antibody titers. Humoral immune response assays (HAI and MN) were performed by Clinical Laboratory Services of Q² Solutions.

A seropositive participant was regarded as a person whose antibody titer was greater than or equal to the cutoff value (=10 1/dilution [DIL]). Seroconversion for HAI was defined as a postvaccination antibody titer of ≥40 1/DIL in the serum of participants who were seronegative before vaccination (i.e., titer <10 1/DIL on day 1). For seropositive participants (i.e., titer ≥10 1/DIL on day 1), seroconversion required a 4-fold increase in postvaccination HAI antibody titer (but ≥40 1/DIL). The seroconversion rate (SCR) was defined as the percentage of participants who had seroconversion after vaccination. The seroprotection rate (SPR) for HAI was defined as the percentage of participants with an HAI antibody titer of ≥40 1/DIL. Geometric mean titers (GMTs) were defined as the antilog of the mean of the log_10_-transformed inverse titers.

The vaccine response for MN was defined as a ≥4-fold increase in the antibody titer from baseline (day 1, prevaccination). Antibody titers below the cutoff of the assay were assigned an arbitrary value of half the cutoff for calculating the vaccine response. The vaccine response rate (VRR) for MN was defined as the percentage of participants with a vaccine response. The mean geometric increase (MGI) was defined as the geometric mean of the within-participant ratios of the postvaccination to prevaccination reciprocal HAI titers. For the MN titer, the seropositivity rate and GMT were derived using an approach similar to that used for assessing HAI.

**Supplementary Text 3**

Participants documented solicited administration site and systemic events using diary cards from day 1 to day 7 after each vaccination. Additionally, the participants reported unsolicited adverse events and instances of medically attended events (MAEs) on diary cards from day 1 to day 21 after each vaccination. Adverse events were graded by severity, from 1 (mild) to 3 (severe). Grade 3 was defined as “significant pain at rest, preventing everyday activities” for pain, “surface diameter >100 mm” for redness and swelling, temperatures of “≥39.0°C (≥102.2°F)” for fever, and “preventing normal activities” for all other solicited and unsolicited events. Safety assessments included monitoring and recording of solicited and unsolicited adverse events, MAEs, potentially immune-mediated diseases, and serious adverse events.

**Supplementary Text 4**

**Statistical Methods**

The sample size was based on whether vaccination with a monovalent H7N9 virus vaccine elicited an HAI antibody response to the vaccine-homologous virus that met or exceeded the [US Food and Drug Administration’s Center for Biologics Evaluation and Research (CBER](file:///C:\Users\ro725208\AppData\Local\Temp\0370180e-ad01-4140-bf20-da540cddd1fa_Gupta_feedback.zip.1fa\Gupta_Vinay_Author_Internal_\Comments_vkg.docx#CBER)) immunogenicity criteria at the day 43 visit. With 60 participants per antigen/adjuvant/age group, the sample size was 840. A dropout/protocol violation rate of not more than 5% yields an approximate sample size of 57 evaluable participants per group, yielding an 82% overall power to fulfill the CBER immunogenicity criteria for SPR and SCR in any one of the adjuvanted vaccine intervention groups, assuming that the reference rates for SPR and SCR, respectively, are 91% and 80% for participants aged 18–64 years and 86% and 75% for participants aged ≥65 years. The 2-sided exact Clopper-Pearson 99.17% CI for SCR or SPR allows parallel assessment of the 6 antigen/adjuvant combinations, addressing multiple comparisons using a Bonferroni adjustment by dividing the 5% type I error (2-sided tests) equally in 6 ways.

Immunogenicity endpoints were analyzed by intervention group and age group in the per-protocol set (PPS). The PPS included participants who had received any study vaccination and had postvaccination immunogenicity data with no major protocol deviations leading to exclusion on day 43 (for the primary endpoint) or on day 22 (for the secondary endpoints). SCR and SPR were calculated along with Clopper‑Pearson exact 2-sided CIs. For day 43, 99.17% CIs were calculated for SCR and SPR. For day 1 (SPR only) and day 22 (SCR and SPR) and placebo (including day 43), 95% CIs were calculated. The GMT, geometric standard deviation, and 95% CI for GMT were calculated as the antilogarithm of the mean of the log_10_ titer values, antilogarithm of the standard deviation, and antilogarithm of the 95% CIs for the mean of the log_10_ titer values, respectively. MGI was calculated as the antilogarithm of the mean of the change from baseline of log_10_ titer values at the postbaseline visit. The percentage of seropositive participants for HAI and MN, the VRR for MN, and the associated Clopper‑Pearson exact 2‑sided 95% CIs were computed. Number and percentage of participants who reported solicited administration site and solicited systemic adverse events within 7 days of each vaccination and unsolicited adverse events within 21 days of each vaccination were presented by intervention group and age group for all participants who received at least 1 dose of the study vaccine.

**Blinding**

Data were collected in an observer-blinded manner. The vaccine was prepared and administered by qualified study personnel who did not participate in data collection, evaluation, review, or evaluation of any study endpoint. The study personnel remained blinded to each participant’s assigned study intervention throughout the study period.
